# Supplementary material for: Multi‐dataset identification of innovative feature genes and molecular mechanisms in keratoconus
Source: J Cell Mol Med. 2024 Sep 19;28(18):e70079. doi: 10.1111/jcmm.70079 (PMC11412914; doi:10.1111/jcmm.70079)
Supplement: Supplementary file 2 — Material S2. [file JCMM-28-e70079-s002.zip › 7.GSEA analysis/3.DBNDD1/KEGG_VALINE_LEUCINE_AND_ISOLEUCINE_DEGRADATION.html]

Details for gene set KEGG\_VALINE\_LEUCINE\_AND\_ISOLEUCINE\_DEGRADATION[GSEA]

|  || Dataset | DBNDD1.group.cls#h\_versus\_l |
| Phenotype | group.cls#h\_versus\_l |
| Upregulated in class | h |
| GeneSet | KEGG\_VALINE\_LEUCINE\_AND\_ISOLEUCINE\_DEGRADATION |
| Enrichment Score (ES) | 0.14893726 |
| Normalized Enrichment Score (NES) | 0.5420097 |
| Nominal p-value | 0.9602386 |
| FDR q-value | 0.9774371 |
| FWER p-Value | 1.0 |
Table: GSEA Results Summary

  

Fig 1: Enrichment plot: KEGG\_VALINE\_LEUCINE\_AND\_ISOLEUCINE\_DEGRADATION      
 Profile of the Running ES Score & Positions of GeneSet Members on the Rank Ordered List

  

| SYMBOL | TITLE | RANK IN GENE LIST | RANK METRIC SCORE | RUNNING ES | CORE ENRICHMENT || 1 | HMGCS2 | na | 275 | 0.517 | 0.1489 | Yes |
| 2 | IL4I1 | na | 3376 | 0.237 | 0.1314 | No |
| 3 | ACAA2 | na | 5976 | 0.151 | 0.1023 | No |
| 4 | BCAT1 | na | 6012 | 0.150 | 0.1469 | No |
| 5 | HMGCL | na | 7626 | 0.111 | 0.1342 | No |
| 6 | ACAA1 | na | 9690 | 0.076 | 0.0977 | No |
| 7 | BCKDHB | na | 10500 | 0.064 | 0.0939 | No |
| 8 | ACAD8 | na | 10516 | 0.064 | 0.1130 | No |
| 9 | DBT | na | 10805 | 0.061 | 0.1231 | No |
| 10 | DLD | na | 11443 | 0.053 | 0.1207 | No |
| 11 | MCCC2 | na | 11666 | 0.050 | 0.1296 | No |
| 12 | BCAT2 | na | 12342 | 0.044 | 0.1234 | No |
| 13 | ABAT | na | 12350 | 0.044 | 0.1366 | No |
| 14 | ALDH3A2 | na | 12835 | 0.040 | 0.1346 | No |
| 15 | MCEE | na | 13027 | 0.038 | 0.1406 | No |
| 16 | PCCB | na | 13599 | 0.033 | 0.1342 | No |
| 17 | ALDH6A1 | na | 14027 | 0.030 | 0.1310 | No |
| 18 | ECHS1 | na | 14374 | 0.027 | 0.1293 | No |
| 19 | ACAT1 | na | 14381 | 0.027 | 0.1374 | No |
| 20 | ACADM | na | 14617 | 0.026 | 0.1384 | No |
| 21 | HIBCH | na | 14851 | 0.024 | 0.1390 | No |
| 22 | ACAT2 | na | 15183 | 0.021 | 0.1359 | No |
| 23 | AOX1 | na | 15596 | 0.018 | 0.1297 | No |
| 24 | OXCT1 | na | 15786 | 0.017 | 0.1295 | No |
| 25 | HADHB | na | 16184 | 0.015 | 0.1224 | No |
| 26 | HMGCS1 | na | 16203 | 0.014 | 0.1263 | No |
| 27 | HADHA | na | 17010 | 0.009 | 0.1059 | No |
| 28 | HADH | na | 17746 | 0.005 | 0.0862 | No |
| 29 | ALDH7A1 | na | 18637 | -0.002 | 0.0610 | No |
| 30 | HSD17B10 | na | 20052 | -0.013 | 0.0243 | No |
| 31 | ACADSB | na | 20458 | -0.017 | 0.0178 | No |
| 32 | PCCA | na | 21630 | -0.028 | -0.0074 | No |
| 33 | MCCC1 | na | 21805 | -0.030 | -0.0033 | No |
| 34 | ALDH1B1 | na | 22410 | -0.036 | -0.0097 | No |
| 35 | ALDH9A1 | na | 22786 | -0.041 | -0.0081 | No |
| 36 | ACADS | na | 23379 | -0.048 | -0.0107 | No |
| 37 | BCKDHA | na | 24729 | -0.067 | -0.0293 | No |
| 38 | HIBADH | na | 25085 | -0.072 | -0.0177 | No |
| 39 | AUH | na | 25234 | -0.074 | 0.0005 | No |
| 40 | IVD | na | 27199 | -0.112 | -0.0220 | No |
| 41 | EHHADH | na | 30010 | -0.189 | -0.0457 | No |
| 42 | OXCT2 | na | 31743 | -0.251 | -0.0194 | No |
| 43 | ALDH2 | na | 33235 | -0.347 | 0.0430 | No |
Table: GSEA details [plain text format]

  

Fig 2: KEGG\_VALINE\_LEUCINE\_AND\_ISOLEUCINE\_DEGRADATION      
 Blue-Pink O' Gram in the Space of the Analyzed GeneSet

  

Fig 3: KEGG\_VALINE\_LEUCINE\_AND\_ISOLEUCINE\_DEGRADATION: Random ES distribution      
 Gene set null distribution of ES for **KEGG\_VALINE\_LEUCINE\_AND\_ISOLEUCINE\_DEGRADATION**
